# Iodine Nutrition and Iodine Supplement Initiation in Association with Thyroid Function in Mildly-to-Moderately Iodine-Deficient Pregnant and Postpartum Women

**DOI:** 10.1093/jn/nxab224

**Published:** 2021-07-13

**Authors:** Synnøve Næss, Maria W Markhus, Tor A Strand, Marian Kjellevold, Lisbeth Dahl, Ann-Elin M Stokland, Bjørn G Nedrebø, Inger Aakre

**Affiliations:** Seafood, Nutrition and Environmental State, Institute of Marine Research, Bergen, Norway; Centre for International Health, Department of Global Public Health and Primary Care, University of Bergen, Bergen, Norway; Seafood, Nutrition and Environmental State, Institute of Marine Research, Bergen, Norway; Centre for International Health, Department of Global Public Health and Primary Care, University of Bergen, Bergen, Norway; Department of Research, Innlandet Hospital Trust, Lillehammer, Norway; Seafood, Nutrition and Environmental State, Institute of Marine Research, Bergen, Norway; Seafood, Nutrition and Environmental State, Institute of Marine Research, Bergen, Norway; Department of Endocrinology, Stavanger University Hospital, Stavanger, Norway; Department of Internal Medicine, Haugesund Hospital, Haugesund, Norway; Department of Clinical Science, University of Bergen, Bergen, Norway; Seafood, Nutrition and Environmental State, Institute of Marine Research, Bergen, Norway

**Keywords:** iodine, thyroid hormones, pregnancy, iodine supplementation, iodine deficiency

## Abstract

**Background:**

Whereas the adverse effects of severe iodine deficiency during pregnancy are well documented, the effects of mild-to-moderate deficiency are not well established.

**Objectives:**

We aimed to explore whether iodine nutrition and timing of iodine supplement initiation are associated with thyroid function in pregnant and postpartum women.

**Methods:**

In this cohort study, 137 pregnant women were enrolled and followed up at gestational weeks (GWs) 18 and 36, and 3 and 6 mo postpartum. Thyroid function tests [thyroid-stimulating hormone (TSH), free triiodothyronine (fT3), and free thyroxine (fT4)], urinary iodine and creatinine concentration (UIC:Cr), and iodine intake (including iodine supplement use) were measured at each time point. The associations between thyroid hormone concentrations and UIC:Cr, iodine intakes, and iodine supplement use were estimated using multiple generalized estimating equation models.

**Results:**

The median UIC at GW18 was 94 μg/L, indicating mild-to-moderate iodine deficiency. UIC:Cr (β; 95% CI) per 100 μg/g was negatively associated with fT3 (−0.191; −0.331, −0.051) and fT4 (−0.756; −1.372, −0.141) concentrations. Iodine intake (β; 95% CI) per 100 μg/d was positively associated with TSH (0.099; 0.022, 0.177), and negatively associated with fT3 (−0.084; −0.0141, −0.027) and fT4 (−0.390; −0.599, −0.182) concentrations. Compared with no use of supplement, those initiating an iodine-containing supplement prepregnancy and continuing through pregnancy had lower TSH (estimated means) (1.35 compared with 1.68 mIU/L, *P* = 0.021), and higher fT3 (4.48 compared with 4.28 pmol/L, *P* = 0.035) and fT4 (15.2 compared with 14.4 pmol/L, *P* = 0.024) concentrations.

**Conclusions:**

Lower iodine availability during pregnancy and postpartum was associated with lower TSH, and higher fT3 and fT4 concentrations. The use of an iodine-containing supplement that was initiated prepregnancy and continuing through pregnancy was associated with lower TSH, and higher fT3 and fT4 concentrations, which may suggest improved thyroid function. These findings support the notion that optimization of iodine intake should start before pregnancy.

This trial was registered at clinicaltrials.gov as NCT02610959.

## Introduction

Sufficient iodine intake is vital for normal thyroid function through its incorporation in the thyroid hormones, triiodothyronine (T3) and thyroxine (T4), which further are important for fetal neurodevelopment (1). Iodine requirements increase during pregnancy and postpartum owing to increased thyroid hormone synthesis, transfer of iodine to the fetus, increased glomerular filtration and urinary iodine losses, and secretion into breast milk during lactation (2). Thus, pregnant and lactating women are groups vulnerable to iodine deficiency and further disturbances of thyroid function (3).

The WHO defines insufficient iodine intake in pregnant and lactating women as median urinary iodine concentration (UIC) <150 and 100 μg/L (4). Mild-to-moderate iodine deficiency in pregnant women can be defined as median UIC in the range of 50–149 μg/L, and severe iodine deficiency as median UIC <50 μg/L (5). Whereas the adverse effects of severe iodine deficiency during pregnancy are well documented (1), the consequences of mild-to-moderate iodine deficiency are less clear and it is uncertain at which level of iodine status the thyroid hormones are affected (6). Several observational studies have reported an association between mild-to-moderate iodine deficiency during pregnancy and impaired child development (7–11). However, these studies do not report data on maternal thyroid function; thus, the mechanisms between mild-to-moderate iodine deficiency and disturbed thyroid function, and, further, impaired child development are not fully explained (12). Of the few published studies, some have found an association between mild-to-moderate iodine deficiency and thyroid function in pregnant and postpartum women (13–15), whereas others have not (16–20).

The benefits of iodine supplementation in mildly-to-moderately iodine-deficient pregnant women remain unclear, even though it is recommended in several parts of the world. A recent systematic review and meta-analysis concluded that there was inconsistent evidence that iodine supplementation improved maternal thyroid function in this group (21). This was also the conclusion in 2 previous systematic reviews (22, 23) and a Cochrane review from 2017 (24). In addition, some studies indicate that initiating an iodine supplement after conception may be too late, and that an abrupt increase in iodine intake might have negative effects on thyroid function which further may harm the developing fetus (14, 25).

The main aims of this article were to explore whether iodine nutrition (UIC and iodine intake) and timing of iodine supplement initiation were associated with altered thyroid function in mildly-to-moderately iodine-deficient pregnant and postpartum women.

## Methods

### Study design

This was a cohort study where a total of 137 pregnant women were enrolled and followed up at gestational weeks (GWs) 18 and 36, and 3 and 6 mo postpartum. The current investigation is a secondary analysis from the study “Mommy's Food” (NCT02610959), which is a 2-armed randomized controlled trial (RCT) where pregnant women were randomly assigned to either receive dietary Atlantic cod (*Gadus morhua*) or continue with their habitual diet during the second and third trimesters of pregnancy (GWs 20–36) (26). The intervention increased the median UIC in the intervention group but had no effects on thyroid function (27). Therefore, in the current article, we wanted to explore potential associations between markers of iodine nutrition (UIC and iodine intake) and thyroid function tests in an observational design. Thus, participants from both the intervention and the control arms of the study were included in a secondary analysis of the data. The number of participants recruited to the study was based on the power calculation for the primary outcome of the study (UIC after the intervention) (26). Because this article was a secondary analysis of the data we did not perform a post hoc power analysis, because this is not recommended (28, 29).

The study visits during pregnancy (GWs 18 and 36) and postpartum (3 and 6 mo) included the collection of biological samples (blood and urine samples) and questionnaires regarding demography, dietary intake, and supplement use. Further information about the study, including study design, power calculation, enrolment, and study procedures, has been published elsewhere (26, 27).

### Participants and recruitment

Participants were recruited through the Women's Clinic at Haukeland University Hospital in Health Region West in Norway from January 2016 to February 2017. In addition, study information was broadcast online through social media and an online magazine for pregnant women in Norway. Inclusion criteria were first-time pregnant, singleton pregnancy, GW ≤19, and Norwegian speaking and/or able to understand Norwegian writing (because of the questionnaires and validated tests of the child being in Norwegian). Exclusion criteria were diseases known to affect iodine status (hypothyroidism, hyperthyroidism, Graves disease, thyroiditis, and thyroid nodules) and fish allergies.

### Outcomes

#### Thyroid function tests

TSH, free triiodothyronine (fT3), and free thyroxine (fT4) were measured in serum samples collected in GWs 18 and 36, and 3 and 6 mo postpartum. Venous blood samples for serum preparation were collected in BD Vacutainer^®^ SST™ vials II Advanced (Becton, Dickinson and Co.) and set to coagulate for a minimum of 30 min before centrifuging (1000–3000 × *g*, room temperature, 10 min) within 60 min after venipuncture. Postseparation, serum samples were stored at −80°C pending analysis at Fürst Medical Laboratory in Oslo, Norway. The serum samples were stored for a maximum of 3 mo before analysis. TSH, fT4, and fT3 were analyzed in serum using magnetic separation and detection by chemiluminescence, labeled with acridinium ester, on an Advia Centaur XPT Immunoassay system (Siemens Healthcare Diagnostics Inc.). For TSH, fT3, and fT4 the analytical CV was 3.1%, 3.3%, and 4.6%, respectively.

For TSH, reference values from MoBa (the Norwegian Mother, Father and Child Cohort Study) were used for pregnant women (GWs 18 and 36) [2.5–97.5 percentiles in *n* = 2577 thyroid peroxidase antibody (TPOAb)-negative pregnant women (mean GW = 18.5): 0.39–2.70 mIU/L] (14). Postpartum, reference values from the Norwegian HUNT Study (The Trøndelag Health Study) were used (2.5–97.5 percentiles in *n* = 514 TPOAb-negative females <40 y old: 0.37–3.30 mIU/L) (30). The total reference population in the HUNT Study consisted of *n* = 17,824 TPOAb-negative women (2.5–97.5 percentile: 0.48–3.60 mIU/L). For fT3 and fT4, the reference values for adults from Fürst Medical Laboratory were used because pregnancy or trimester-specific reference ranges were not available (fT3: 3.5–6.5 pmol/L; fT4: 11.0–23.0 pmol/L) (31).

Thyroid dysfunction was defined by thyroid function tests outside reference ranges (32). Overt hypothyroidism was defined as TSH above and fT4 below reference values. Overt hyperthyroidism was defined as TSH below and fT4 above reference values. Subclinical hypothyroidism and hyperthyroidism were defined as TSH above and below reference values, respectively, and normal fT4 values. Isolated hypothyroxinemia was defined as normal TSH values and fT4 values below reference values.

#### UIC and urinary creatinine concentrations

In GWs 18 and 36, the participants collected spot urine samples for 6 consecutive days (6 spot urine samples). Equal amounts of urine from the 6 spot urine samples were homogenized into 1 pooled composite sample of 1 mL. At 3 and 6 mo postpartum 1 spot urine sample was collected from the participants. The participants were instructed to collect the spot urine sample between 16:00 and midnight, to reduce the within-day variation between samples. Urine samples were stored at −20°C in cryotubes (CryoTube^TM^ Vials Nunc; Thermo Fischer Scientific) pending analysis. Iodine concentration in the urine samples was determined by inductively coupled plasma mass spectrometry (ICP-MS). Before analysis, the urine samples were defrosted in a refrigerator, diluted with 1% tetramethylammonium hydroxide, and filtered using a sterile membrane filter with a 0.45-μg pore size and single-use syringe. Samples were analyzed by an Agilent 7500 via ICP-MS at the Institute of Marine Research (IMR), Bergen, Norway. Samples were analyzed against a urine calibration curve (standard addition curve). Certified reference material was used to check the internal validity of the method: Seronorm Trace Elements Urine (Nycomed Pharma) [iodine content: 84 μg/L (range: 72–96 μg/L) and 304 μg/L (range: 260–348 μg/L)]. The measurement uncertainty of the method has been assessed based on internal reproducibility and analysis of standard reference material, and is set at 20% in the entire range (2–297 μg/L).

Urinary creatinine concentration was analyzed using a MAXMAT PL II multidisciplinary diagnostic platform with a creatinine PAP kit (ERBA Diagnostics). The urine samples were defrosted at room temperature and centrifuged in an Eppendorf (5810R) centrifuge (15 min, 2000 ×* g*, and 4°C). An aliquot of 200 μL was transferred to the test tube and placed in the MAXMAT carousel for analysis. The method was calibrated with 1 standard and further controlled with 2 independent controls.

The reference values from the WHO of UIC during pregnancy (150 μg/L) and lactation (100 μg/L) were used to assess adequate iodine status (33). Mild-to-moderate iodine deficiency was defined as median UIC 50–149 μg/L (5). UIC was presented as UIC:creatinine ratio (UIC:Cr) (μg/g) in the models to adjust for individual hydration status (34) and, owing to better association with iodine intake from the FFQ, compared with only UIC (**Supplemental Table 1**).

#### Iodine intake and iodine supplement use

Iodine intake and use of iodine supplements were self-reported and estimated from a validated electronic iodine-specific FFQ which was developed specifically for this study (35). The FFQ was completed by the participants at GWs 18 and 36, and 3 and 6 mo postpartum. In the FFQ completed in GW 18, the participants were asked to report an estimate of their diet since they became pregnant, whereas the FFQ in GW 36 covered the intake during the last 16 wk (approximately since the last time they completed the FFQ). The FFQs completed 3 and 6 mo postpartum were intended to cover the participants’ diet during the past 3 mo. The FFQ consisted of 60 iodine-rich food items (milk and dairy products, fish and other seafood, and eggs) and the use of dietary supplements. Total iodine intake (μg/d) was summarized and estimated from the food items and dietary supplements. More information regarding the FFQ and calculation of iodine intake has been published elsewhere (35).

The use of iodine-containing supplements was reported by the participants at each specific time point. In addition, the use of prepregnancy iodine-containing supplements was reported in the FFQ completed in GW 18. The use of iodine-containing dietary supplements was categorized to a dichotomous variable and defined as >2 times/wk (yes/no). Further, the timing of iodine supplement initiation, from prepregnancy and until GW 18, was merged and categorized into the following categories:

*None*: no use reported either prepregnancy or GW 0–18;
*Prepregnancy*: only reported use prepregnancy;
*GW 0–18*: only reported use GW 0–18;
*Prepregnancy and GW 0–18*: reported use, both prepregnancy and GW 0–18.

The amount of iodine in the iodine-containing supplements varied from 75 to 225 μg per recommended daily dose according to the product label, with most supplements containing 150–200 μg I/dose.

The recommended intake amount from the WHO of 250 μg/d for pregnant and lactating women was used to assess sufficient iodine intake (4).

#### Background variables

Baseline characteristics from the study participants were retrieved from the electronic questionnaires conducted in GW 18. The questionnaires included information regarding pregnancy week, age, education level, prepregnancy weight and height, nicotine use during pregnancy, and use of prescribed medications during the last 6 wk. Prepregnancy BMI (in kg/m^2^) was calculated by prepregnancy weight (kg) divided by the square of the height in meters. The ferritin concentration was analyzed in serum samples and collected as described for the aforementioned thyroid function tests. Serum ferritin was analyzed by an immunoturbidimetric method using an Advia Chemistry XPT system (Siemens Medical Solutions Diagnostica). The CV for serum ferritin concentration was 2.5%.

### Ethics

The study was approved by the Regional Committee for Medical and Health Research Ethics West (REK 2015/879). The trial complies with the Declaration of Helsinki and written informed consent was obtained from the participants after giving both written and oral information about the study. The participants could withdraw from the study at any time without giving any reason.

### Statistics

Variables were tested for normality by visual inspection of Q-Q plots and histograms. Descriptive results are reported as proportions (%) for categorical variables. For continuous variables means ± SDs, medians, or percentiles are reported as appropriate. *P* values < 0.05 were considered statistically significant.

We measured the associations between the thyroid hormones (TSH, fT3, and fT4) and UIC:Cr, UIC, and iodine intake in generalized estimating equations (GEEs), including data from all time points (GWs 18 and 36, and 3 and 6 mo postpartum). In the GEE analyses, the associations between the variables of the model at different time points were analyzed concurrently, and the interdependence in multiple data from each participant at the different time points was accounted for (36). TSH, fT3, fT4, or thyroid dysfunction (dichotomized) were used as dependent variables, and UIC:Cr, UIC, or iodine intake as either continuous or dichotomous independent variables. In the GEE models, we used an exchangeable correlation matrix, and for the continuous outcome variables (TSH, fT3, and fT4) we used the Gaussian distribution family with identity link functions. In the analyses with dichotomous outcome variables (thyroid dysfunction), we used a binomial distribution with logit link functions. The concentrations of TSH were log2 transformed in the analyses to achieve normality.

Potential covariates in adjusted models were included using “Purposeful selection of covariates” as suggested by Hosmer et al. (37). The following covariates to use were considered based on known associations from the literature: age of the mother, prepregnancy BMI, education level, nicotine use during pregnancy, and ferritin concentration. All potential covariates were assessed in univariate models with the outcome variable and further included if the *P* value was <0.25. In the multivariable model containing all covariates identified at step 1 (*P* < 0.25), variables that were not significant at the traditional *P* < 0.05 level were excluded stepwise if the coefficient for UIC:Cr, UIC, or iodine intake did not change considerably (>20%). The following covariates (adjustment variables) were included in the GEE models: TSH: no covariates; fT3, fT4, and fT4:fT3 ratio: prepregnancy BMI and ferritin concentrations; and thyroid dysfunction: prepregnancy BMI.

The correlation of thyroid hormones with UIC:Cr, UIC, and iodine intake was assessed using Spearman's rank order correlation coefficient (Spearman's ρ).

Statistical analyses were performed using Statistical Package for the Social Sciences (SPSS) for Windows, version 26 (IBM Corporation).

## Results

**FIGURE 1:**
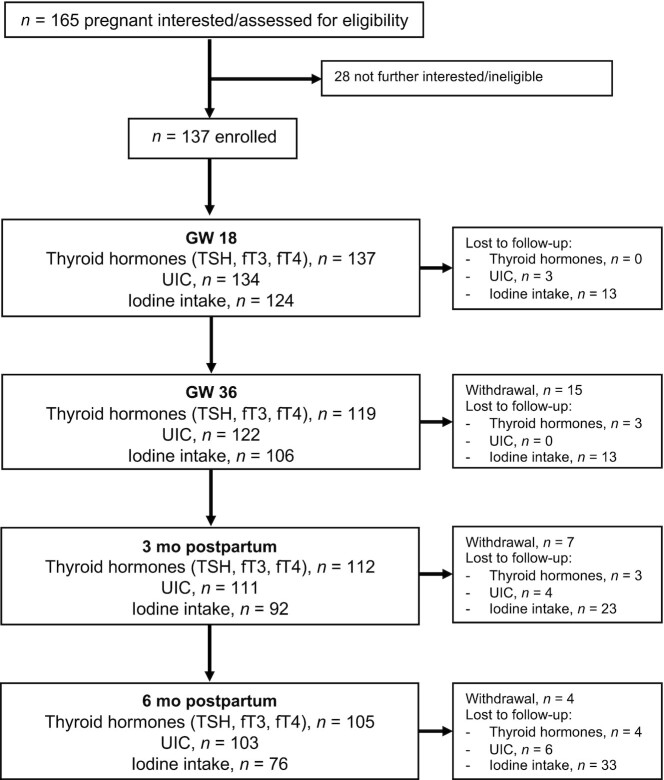
Flowchart of the study population and data available at each time point. fT3, free triiodothyronine; fT4, free thyroxine; GW, gestational week; TSH, thyroid-stimulating hormone; UIC, urinary iodine concentration.

**Figure 1** gives the flow of the study population and data used in this study. A total of 137 pregnant women were enrolled in the study at GW 18. **Table 1** shows baseline characteristics of the study population.

**TABLE 1:** Baseline characteristics of pregnant women (GW 18) enrolled in the Mommy's Food study^1^

Characteristic	*n*	Value
Age, y	135	29.3 ± 3.4
GW	127	19.0 ± 1.3
Prepregnancy BMI, kg/m^2^	132	22.2 [20.6–24.3]
Education level	133	
Elementary school		2 (1.5)
High school		17 (13)
≤4 y university/college		33 (25)
>4 y university/college		81 (61)
Nicotine use ≤GW 8^2^	132	
Smoking		1 (0.8)
Snuff		12 (9)

1 Values are means ± SDs, medians [IQRs], or *n* (%). GW, gestational week.

2 No participants reported use of nicotine after GW 8.

**Table 2** shows the thyroid hormones (TSH, fT3, and fT4), UIC, UIC:Cr, and iodine intake during pregnancy (GWs 18 and 36) and postpartum (3 and 6 mo). The median UIC was below the recommended WHO concentrations (150 μg/L during pregnancy and 100 μg/L during lactation) at all time points, with a median UIC of 94, 85, 74, and 84 μg/L at the 4 time points, respectively. Thus, this indicated mild-to-moderate iodine deficiency in this population group. The estimated iodine intake was below the WHO recommended amount of iodine intake during pregnancy and lactation (250 μg/d) at all time points, with a median iodine intake of 202, 153, 143, and 134 μg/d, respectively. In GW 18, 41% of the participants took an iodine-containing supplement. This decreased to 28% in GW 36, and 30% and 17% at 3 and 6 mo postpartum, respectively. A total of 25% of the participants took an iodine-containing supplement prepregnancy.

**TABLE 2:** Thyroid hormones (TSH, fT3, and fT4), UIC, and iodine intake in pregnant and postpartum women^1^

	Time point							
	GW 18		GW 36		3 mo postpartum		6 mo postpartum	
Variable	*n*	Value	*n*	Value	*n*	Value	*n*	Value
TSH, mIU/L								
Median [IQR]	137	1.4 [1.0–2.1]	119	1.7 [1.3–2.2]	112	1.3 [0.82–1.6]	105	1.3 [0.75–1.7]
p10–p90		0.84–2.5		0.9–3.0		0.54–2.2		0.49–2.7
p2.5–p97.5		0.26–3.4		0.47–3.9		0.035–2.9		<0.01–6.8
fT3, pmol/L								
Median [IQR]	137	4.3 [3.9–4.6]	119	3.9 [3.6–4.1]	112	4.6 [4.3–4.9]	105	4.7 [4.4–5.0]
p10–p90		3.7–4.8		3.5–4.4		4.1–5.4		4.1–5.4
p2.5–p97.5		3.5–5.3		3.3–4.8		3.8–5.9		3.7–9.5
fT4, pmol/L								
Median [IQR]	137	13.8 [12.9–14.9]	119	13.4 [12.4–14.4]	112	15.3 [14.2–17.1]	105	15.5 [14.4–17.3]
p10–p90		11.9–16.2		11.3–15.3		13.5–18.6		13.4–18.4
p2.5–p97.5		11.3–17.0		9.9–16.6		12.4–22.6		12.5–26.7
Thyroid dysfunction, *n* (%)								
Overt hypothyroidism	137	1 (0.7)	119	2 (1.7)	112	2 (1.8)	105	1 (1.0)
Subclinical hypothyroidism		7 (5.1)		11 (9.2)		0		6 (5.7)
Overt hyperthyroidism		0		0		1 (0.9)		3 (2.9)
Subclinical hyperthyroidism		5 (3.6)		2 (1.7)		4 (3.6)		5 (4.8)
Isolated hypothyroxinemia		0		5 (4.2)		1 (0.9)		0
UIC,^2^ μg/L								
Median [IQR]	134	94 [62–130]	122	85 [57–123]	111	74 [42–130]	103	84 [49–120]
Mean ± SD		103 ± 56		98 ± 55		97 ± 79		97 ± 79
UIC:creatinine ratio,^2^ μg/g								
Median [IQR]	134	104 [81–144]	122	104 [82–155]	111	69 [40–107]	103	69 [50–96]
Mean ± SD		116 ± 51		124 ± 58		85 ± 64		77 ± 38
Total iodine intake,^3^ μg/d								
Median [IQR]	124	202 [106–275]	106	153 [119–253]	92	143 [83–240]	76	134 [79–201]
Mean ± SD		202 ± 108		180 ± 87		166 ± 101		154 ± 95
<250 μg/d, %		61		75		79		80
≥250 μg/d, %		39		25		21		20
Iodine intake from supplements,^4^ μg/d								
Mean ± SD (all)	124	69 ± 81	106	42 ± 72	92	50 ± 72	76	30 ± 62
Mean ± SD (only supplement users)		163 ± 24		172 ± 22		158 ± 34		161 ± 30
Supplement user, yes, %		41		28		30		17
Supplement user, no, %		59		72		70		83

1 fT3, free triiodothyronine; fT4, free thyroxine; GW, gestational week; TSH, thyroid-stimulating hormone; UIC, urinary iodine concentration.

2 Pooled sample of 6 spot urine samples from 6 consecutive days in GWs 18 and 36. One spot urine sample at 3 and 6 mo postpartum.

3 Estimated total iodine intake from foods and supplements from a validated iodine-specific FFQ (35).

4 Supplement user defined as taking iodine-containing supplements >2 times/wk.

**Table 3** gives the associations of thyroid hormones with UIC:Cr and iodine intake in GEE models. UIC:Cr was negatively associated with fT3 and fT4 concentrations, showing an adjusted coefficient (95% CI) (per 100 μg/L) of −0.191 (−0.331, −0.051) (*P* = 0.008) and −0.756 (−1.372, −0.142) (*P* = 0.016), respectively. These associations were not seen for UIC alone, only when using UIC:Cr (**Supplemental Table 2**). No association was seen between TSH or thyroid dysfunction and UIC:Cr, nor UIC. No association was seen between fT4:fT3 ratio and UIC:Cr, but fT4:fT3 ratio was negatively associated with UIC with an adjusted coefficient (per 100 μg/L) of −0.065 (95% CI: −0.115, −0.015) (*P* = 0.011) (Supplemental Table 2). TSH was positively associated with iodine intake, showing an adjusted coefficient (per 100 μg/d) of 0.099 (95% CI: 0.022, 0.177) (*P* = 0.012). Furthermore, fT3 and fT4 were negatively associated with iodine intake, showing an adjusted coefficient (per 100 μg/d) of −0.084 (95% CI: −0.141, −0.027) (*P* = 0.004) and −0.390 (95% CI: −0.599, −0.182) (*P* < 0.001), respectively. We found no associations of fT4:fT3 ratio or thyroid dysfunction with iodine intake (Table 3).

**TABLE 3:** Associations of thyroid function tests (TSH, fT3, and fT4) and disturbed thyroid function with repeated measurements of UIC:creatinine ratio and iodine intake during pregnancy (GWs 18 and 36) and postpartum (3 and 6 mo) in GEE models^1^

	Independent variables							
	UIC:creatinine ratio^2^				Iodine intake^3^			
	Unadjusted		Adjusted		Unadjusted		Adjusted	
Dependent variables	Coefficient (95% CI)	*P*	Coefficient (95% CI)	*P*	Coefficient (95% CI)	*P*	Coefficient (95% CI)	*P*
TSH^4^	0.041 (−0.159, 0.240)	0.690	0.041 (−0.159, 0.240)	0.690	0.099 (0.022, 0.177)	0.012	0.099 (0.022, 0.177)	0.012
fT3^5^	−0.206 (−0.340, −0.072)	0.003	−0.191 (−0.331, −0.051)	0.008	−0.046 (−0.109, 0.017)	0.156	−0.084 (−0.141, −0.027)	0.004
fT4^5^	−0.791 (−1.365, −0.218)	0.007	−0.756 (−1.372, −0.141)	0.016	−0.325 (−0.537, −0.113)	0.003	−0.390 (−0.599, −0.182)	<0.001
fT4:fT3 ratio^5^	−0.042 (−0.113, 0.029)	0.249	−0.061 (−0.134, 0.013)	0.104	−0.028 (−0.075, 0.020)	0.255	−0.017 (−0.066, 0.032)	0.489
Thyroid dysfunction^6^	1.40 (0.93, 2.12)	0.106	1.36 (0.89, 2.09)	0.158	1.15 (0.83, 1.60)	0.406	1.11 (0.79, 1.58)	0.545

1 GEE models with exchangeable correlation matrix. TSH, fT3, fT4: normal distribution with identity link function. Thyroid dysfunction: binomial distribution with logit link function. UIC:creatinine coefficient expressed as per 100 μg/g. Iodine intake coefficient expressed as per 100 μg/d. fT3, free triiodothyronine; fT4, free thyroxine; GEE, generalized estimating equation; GW, gestational week; TSH, thyroid-stimulating hormone; UIC, urinary iodine concentration.

2 Pooled sample of 6 spot urine samples from 6 consecutive days in GWs 18 and 36. One spot urine sample at 3 and 6 mo postpartum.

3 Estimated total iodine intake (from foods and supplements) from a validated iodine-specific FFQ (35).

4 Log2-transformed values of TSH owing to skewed data. Covariates in adjusted model: none.

5 Covariates in adjusted model of fT3, fT4, and fT4:fT3 ratio: prepregnancy BMI and ferritin concentration.

6 Dichotomous variable: 0 = reference category, normal thyroid function; 1 = disturbed thyroid function (TSH and/or fT4 or fT3 outside reference ranges). Coefficient given as OR (95% CI). Covariates in adjusted model: prepregnancy BMI.

**FIGURE 2:**
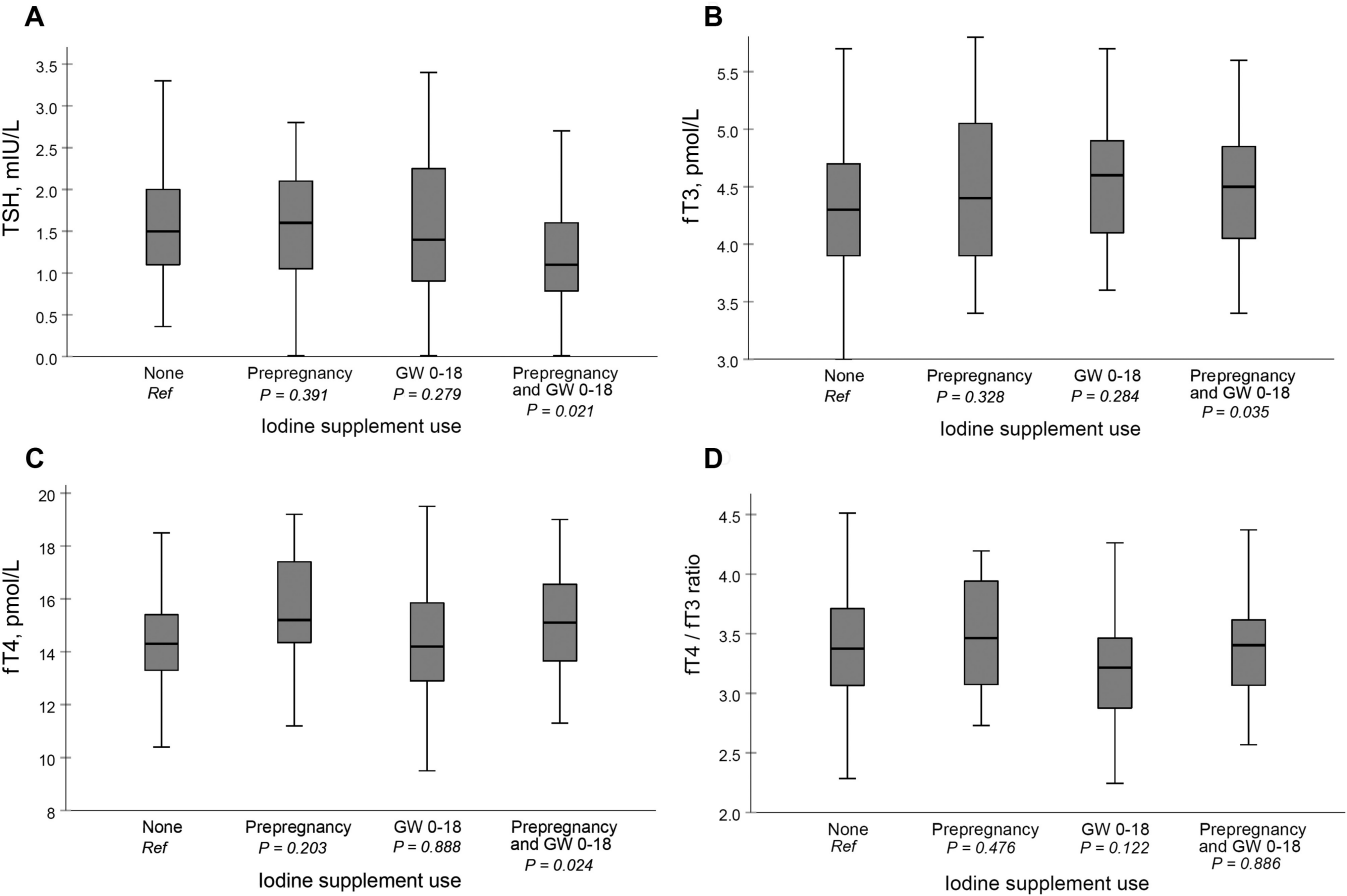
Box plot of TSH (A), fT3 (B), fT4 (C), and fT4:fT3 ratio (D) during pregnancy and postpartum by categories of timing of iodine-containing supplement initiation (from prepregnancy until GW 18). *P* values were obtained from adjusted generalized estimating equation models (Supplemental Tables 3–6). Boxes indicate the upper (75th percentile) and lower (25th percentile) quartiles with the thick black line giving the median (50th percentile). The T-bars indicate 1.5 × length of the box (IQR). fT3, free triiodothyronine; fT4, free thyroxine; GW, gestational week; TSH, thyroid-stimulating hormone.

**Figure 2** and **Supplemental Tables 3**–**6** show concentrations of TSH, fT3, and fT4, and the fT4:fT3 ratio by categories of the timing of iodine supplement initiation (from prepregnancy until GW 18). In total, 15% of the participants reported the use of an iodine-containing supplement both prepregnancy and during the first part of pregnancy (GWs 0–18). Further, 9% reported only use prepregnancy, 23% reported only use during the first part of pregnancy, and 53% reported no use either prepregnancy or during the first part of pregnancy. Compared with no use of an iodine supplement , initiation of an iodine-containing supplement prepregnancy and continuing through pregnancy (GWs 0–18) was in adjusted GEE models associated with lower concentrations of TSH (estimated means: 1.35 compared with 1.68 mIU/L, *P* = 0.021) and higher concentrations of fT3 (4.48 compared with 4.28 pmol/L, *P* = 0.035) and fT4 (15.2 compared with 14.4 pmol/L, *P* = 0.024). This association was not seen for those initiating an iodine-containing supplement after the conception of pregnancy.

## Discussion

In this observational study of pregnant and postpartum women with mild-to-moderate iodine deficiency, we found that iodine nutrition was associated with altered thyroid hormone function. Lower UIC:Cr and iodine intake were associated with higher fT3 and fT4 concentrations, whereas lower iodine intake was associated with lower TSH concentrations. In contrast, the use of an iodine supplement that was initiated before conception and continuing through pregnancy was associated with lower TSH, and higher fT3 and fT4 concentrations.

The women in this study had a median UIC <100 μg/L throughout pregnancy and until 6 mo postpartum, corresponding to mild-to-moderate iodine deficiency (5). In addition, the mean iodine intake was well below the WHO recommendations of 250 μg/d for pregnant and lactating women at all time points. As of today, several countries and health authorities recommend iodine supplementation during pregnancy, owing to frequently observed mild-to-moderate iodine deficiency in this group (21). However, this recommendation is in contrast to the evidence, because there are uncertain data to support the effect of iodine supplementation in this group (21, 24, 38). In this study, we found no effect on thyroid hormone function, either beneficial or harmful, if initiating an iodine-containing supplement after the conception of pregnancy. However, initiation of iodine supplements before conception and continuing through pregnancy was associated with lower concentrations of TSH and higher concentrations of fT4 and fT3, which might suggest improved thyroid function. Thus, iodine supplementation should start before pregnancy, such that intrathyroidal iodine stores are optimized before conception. Initiation of iodine supplementation too late may also be the reason why other studies that examined the effect of iodine supplementation during pregnancy found mostly no effect on thyroid function (39–43). Currently, the largest RCT with iodine supplementation in mildly-to-moderately iodine-deficient pregnant women was conducted in India and Thailand from 2008 to 2011 (39). Supplementing 200 μg I/d had no effect on maternal thyroid function or child neurodevelopment. However, the stratum consisting of Indian pregnant women had a median UIC at 188 μg/L, indicating iodine sufficiency. When restricting the analysis to the participants from Thailand (baseline median UIC of 112 μg/L) (44), iodine supplementation resulted in a small negative effect on maternal thyroxine (T4) concentrations. There was, however, no effect of iodine supplementation on child development in this subgroup.

In contrast to the positive association between the use of iodine supplements preconception and continuing through pregnancy and fT4 concentrations, we found a negative association of UIC:Cr and iodine intake with fT4 concentrations. This was not expected, because based on physiological adaptations one would rather expect a positive association between iodine status and fT4 concentrations (45, 46). Furthermore, we found a positive association between iodine intake and TSH concentrations. Decreased TSH concentrations may occur in populations with mild-to-moderate iodine deficiency because of an increase in thyroid nodularity and autonomy (47). TSH and fT4 concentrations are usually inversely correlated, which we also found (Supplemental Table 1). Consequently, this may explain the inverse association of UIC:Cr and iodine intake with fT4 concentrations. Similar to our results, an inverse association between iodine nutrition and fT4 concentrations, in pregnant women, was also found in birth cohorts from Sweden (13) and Norway (14). In addition, studies have also shown that women who started taking iodine-containing supplements (16, 25, 48) or used iodized salt (49) during pregnancy had higher TSH concentrations, in line with our observation of a positive association between iodine intake and TSH concentrations. How an increased intake of iodine may affect the thyroid and give an inhibited production of fT4 has been suggested by others (13, 14, 25) to be a result of a “stunning effect” on thyroid hormone production, a similar mechanism to the Wolff–Chaikoff effect (50). The Wolff–Chaikoff effect may occur after acute large doses of iodine and shuts down the thyroid hormone synthesis to prevent an overproduction of thyroid hormones. However, the doses described when the Wolff–Chaikoff effect occurs are much larger than the recommended intake amount of iodine, and at which doses the stunning effect may occur is still uncertain. The stunning effect is proposed to be a result of an abrupt increase in intake; for example, after initiation of an iodine supplement during pregnancy. In this study, we did not have data on prepregnancy UIC or total iodine intake. Thus, we cannot draw any conclusion on how an abrupt increase of iodine intake at the beginning of pregnancy influences thyroid function. Furthermore, the mechanisms of this stunning effect have not been described properly and remain unclear. In addition, several studies also found no effect of an increased iodine intake during pregnancy on thyroid function (39–42). Thus, the evidence of whether an abrupt increase in iodine intake, even within the recommended intake range, during pregnancy can be potentially harmful for thyroid hormone production is still not conclusive.

We observed a negative association of fT4:fT3 ratio with UIC, but not with UIC:Cr and iodine intake; however, the negative coefficients indicated a similar direction of the associations. The negative association with UIC can be explained by the autoregulatory mechanisms when the availability of iodine is low—where the secretion of T3 over T4 is preferred to save 1 iodine atom and to prioritize production of the active hormone (T3) (46). This is also why one typically sees elevated concentrations of fT3 with lower iodine intake (45, 46, 51), which also was confirmed in our study.

There were no clear associations between iodine status and thyroid dysfunction, and the study population had a low prevalence of thyroid dysfunction according to the thyroid function tests (Table 2). This was observed even though the reference range for TSH in pregnancy, derived from the MoBa study to define thyroid dysfunction, was lower than most other reference ranges (32). However, this reference range was the most appropriate, because it is recommended to use population-, trimester-, and assay-specific reference ranges when these are available (52). Also, the effect sizes observed between UIC:Cr and iodine intake with TSH, fT3, and fT4 were relatively low. Thus, the clinical impact of the observed associations between iodine nutrition and thyroid function in this study is not known. However, studies have found that both low and high maternal thyroid function during pregnancy are associated with less child total gray matter and cortex volume (53, 54) and lower child IQ (55). Consequently, the observed associations between maternal iodine nutrition and thyroid function may also have importance for child development, even though the effect sizes were low.

There are some limitations of this study that should be mentioned. The explorative nature of the study limits the assumption of causality. Furthermore, the study comprised a relatively low number of participants, which has implications for its internal validity because of the reduced precision of the effect estimates and reduced statistical power. Low statistical power increases the risk of making type 2 errors. In other words, because of the limited sample size, we could have overlooked clinically meaningful differences. However, despite this, we demonstrated associations between iodine nutrition, supplement use, and thyroid function. Reduced statistical power will generally not result in type 1 errors and we therefore believe that the observed associations were not affected by the relatively small sample size. It is also worth mentioning that each participant had ≤4 repeated measurements of each outcome and exposure, improving the reliability of these measurements. Thus, considering the aforementioned limitations and strengths of the sample size, we believe our data are relevant for similar populations. We did not measure thyroid size, thyroglobulin (Tg), TPOAb, or thyroglobulin antibody (TgAb) which could have added further information regarding thyroid function. Also, breast milk iodine concentration (BMIC) was not included in this study. This is a limitation, because BMIC may be a more accurate marker of iodine status postpartum (56). Thus, the overall UIC status at 3 and 6 mo postpartum should be interpreted with caution. The strengths of this study are the use of 2 markers of iodine nutrition, both UIC and iodine intake. The UIC during pregnancy was derived from spot samples from 6 consecutive days. It has been suggested that ≥10 spot urine samples are needed to reliably estimate individual iodine status (57). Ten samples were not feasible to obtain in this study, but we believe that 6 samples are highly strengthened compared with most other studies, that typically use 1 spot sample, because it reduces the day-to-day variation and further the inter- and intraindividual variability. UIC was also presented as UIC:Cr to adjust for individual hydration status. Because the participants in this study consisted of a rather homogeneous population group—first-time pregnant women with similar age and BMI ranges (Table 1) with a sufficient energy and protein intake—we decided to present the main results as UIC:Cr, which is preferred in a homogeneous population (34). The use of self-reported dietary intake from an FFQ has several limitations, and measurement error may have reduced the power of the reported associations (58). However, the FFQ used in the study was designed specifically to capture iodine intake in this population group, and it has previously been validated (35).

The thyroid can store iodine for ∼3 mo for thyroid hormone production (59), thus the prepregnancy iodine status may be important for coping with the mechanisms of increased demand for thyroid hormone synthesis during pregnancy (60). To date, no RCTs with supplementation starting preconception have been published, and few observational studies have included prepregnancy iodine status. Well-designed RCTs are highly warranted; however, this may not be feasible owing to ethical considerations. Thus, if RCTs are not achievable, well-designed cohort studies with sufficient sample size and including prepregnancy iodine status with appropriate measurements of iodine nutrition (UIC and dietary intake) and thyroid function should be considered.

In conclusion, in an observational study of pregnant and postpartum women with mild-to-moderate iodine deficiency, we found that lower UIC:Cr and iodine intake were associated with higher fT3 and fT4 concentrations, whereas lower iodine intake was associated with lower TSH concentrations. In contrast, the use of an iodine-containing supplement that was initiated before conception and continuing through pregnancy was associated with lower TSH and higher fT3 and fT4 concentrations, which suggest improved thyroid function. Taken together, these findings support the notion that optimization of iodine intake should start before pregnancy.

## Supplementary Material

nxab224_Supplemental_File

## Data Availability

Requests for data collected in the Mommy's Food study (such as deidentified participant data) can be made to the corresponding author, and requests will be considered on an individual basis. Any requests require completion and approval of the application for use of data from the Mommy's Food study. The trial project group will review and, if acceptable and approved by the Regional Committee for Medical and Health Research Ethics West, Norway, provide approval of the request. A signed data-sharing access agreement will be required. The data will be provided as an SPSS data set. Any other format requests might incur costs to the requestor. To facilitate the data access process, please contact MWM at maria.wik.markhus@hi.no and mammasmat@hi.no.

## Notes

Supported by Norwegian Seafood Research Fund (http://www.fhf.no/) grant 901038. The funder had no role in the design of the study, analysis, interpretation of data, or writing of this article.

Author disclosures: The authors report no conflicts of interest.

Supplemental Tables 1–6 are available from the “Supplementary data” link in the online posting of the article and from the same link in the online table of contents at https://academic.oup.com/jn/.

Abbreviations used: BMIC, breast milk iodine concentration; fT3, free triiodothyronine; fT4, free thyroxine; GEE, generalized estimating equation; GW, gestational week; HUNT Study, The Trøndelag Health Study; ICP-MS, inductively coupled plasma mass spectrometry; IMR, Institute of Marine Research; MoBa, the Norwegian Mother, Father and Child Cohort Study; RCT, randomized controlled trial; TPOAb, thyroid peroxidase antibody; TSH, thyroid-stimulating hormone; T3, triiodothyronine; T4, thyroxine; UIC, urinary iodine concentration; UIC:Cr, urinary iodine concentration:creatinine ratio.
