# Supplementary material for: Iodine Nutrition and Iodine Supplement Initiation in Association with Thyroid Function in Mildly-to-Moderately Iodine-Deficient Pregnant and Postpartum Women
Source: J Nutr. 2021 Jul 13;151(10):3187–96. doi: 10.1093/jn/nxab224 (PMC8485914; doi:10.1093/jn/nxab224)
Supplement: nxab224_Supplemental_File [file nxab224_supplemental_file.docx]

## Online Supplementary Material (OSM)

**Supplementary Table 1:** Spearman’s rho correlation coefficients matrix between TSH, fT3, fT4, UIC and iodine intake

**Supplementary Table 2:** Associations between thyroid functions tests (TSH, fT3 and fT4) and disturbed thyroid function with repeated measurements of UIC during pregnancy (GW 18 and 36) and post-partum (3 and 6 months) in generalized estimating equations (GEE) models

**Supplementary Table 3:** TSH (mIU/L) by categories of timing of iodine-containing supplement (from pre-pregnancy until GW 18) in GEE models of repeated measurements during pregnancy (GW 18 and 36) and post-partum (3 and 6 months)

**Supplementary Table 4:** fT3 (pmol/L) by categories of timing of iodine-containing supplement (from pre-pregnancy until GW 18) in GEE models of repeated measurements during pregnancy (GW 18 and 36) and post-partum (3 and 6 months)

**Supplementary Table 5:** fT4 (pmol/L) by categories of timing of iodine-containing supplement (from pre-pregnancy until GW 18) in GEE models of repeated measurements during pregnancy (GW 18 and 36) and post-partum (3 and 6 months)

**Supplementary Table 6:** fT4/fT3 ratio by categories of timing of iodine-containing supplement (from pre-pregnancy until GW 18) in GEE models of repeated measurements during pregnancy (GW 18 and 36) and post-partum (3 and 6 months)

**Supplementary Table 1** Spearman’s rho correlation coefficients matrix between TSH, fT3, fT4, UIC and iodine intake

|  | N | TSH | fT3 | fT4 | UIC | UIC/  Creatinine ^a^ | Iodine intake ^b^ |
| --- | --- | --- | --- | --- | --- | --- | --- |
| TSH | 473 | 1.00 |  |  |  |  |  |
| fT3 | 473 | -0.253  (*P* <0.001) | 1.00 |  |  |  |  |
| fT4 | 473 | -0.299  (*P* <0.001) | 0.472  (*P* <0.001) | 1.00 |  |  |  |
| UIC ^a^ | 464 | -0.045  (*P*= 0.330) | 0.023  (*P*= 0.623) | -0.026  (*P*= 0.579) | 1.00 |  |  |
| UIC/Creatinine ^a^ | 464 | 0.106  (*P*= 0.023) | -0.266  (*P*< 0.001) | -0.234  (*P*< 0.001) | 0.567  (*P*< 0.001) | 1.00 |  |
| Iodine intake ^b^ | 390 | 0.078  (*P*= 0.123) | -0.098  (*P*= 0.054) | -0.142  (*P*= 0.005) | 0.288  (*P* <0.001) | 0.418  (*P* <0.001) | 1.00 |

Data from all time points (pregnancy (GW 18 and 36) and post-partum (3 and 6 months)) included.

^a^ Pooled sample of six spot urine sample from six consecutive days in gestational week 18 and 36. One spot urine sample 3- and 6-months post-partum.

^b^ Estimated total iodine intake (from foods and supplements) from a validated iodine specific food frequency questionnaire (Næss et al. 2019).

**Supplementary Table 2** Associations between thyroid functions tests (TSH, fT3 and fT4) and disturbed thyroid function with repeated measurements of UIC during pregnancy (GW 18 and 36) and post-partum (3 and 6 months) in generalized estimating equations (GEE) models

|  | **Urinary iodine concentration (UIC)** ^a^ | | | |
| --- | --- | --- | --- | --- |
|  | Unadjusted | | Adjusted | |
| **Variables** | Coefficient  (95% CI) | *P* | Coefficient  (95% CI) | *P* |
| **TSH** ^b^ | -0.010  (-0.189, 0.169) | *0.911* | -0.010  (-0.189, 0.169) | *0.911* |
| **fT3** ^c^ | 0.028  (-0.072, 0.127) | *0.583* | 0.029  (-0.084, 0.142) | *0.612* |
| **fT4** ^d^ | -0.164  (-0.544, 0.216) | *0.397* | -0.168  (-0.591, 0.256) | *0.438* |
| **fT4/fT3 ratio** | -0.061  (-0.113, -0.010) | *0.018* | -0.065  (-0.115, -0.015) | *0.011* |
| **Thyroid dysfunction** ^e^ | 1.32  (0.96, 1.82) | *0.091* | 1.30  (0.92, 1.82) | *0.126* |

GEE models with exchangeable correlation matrix. TSH, fT3, fT4: Normal distribution with identity link function. Thyroid dysfunction: Binomial distribution with logit link function. UIC coefficient given in per 100 µg/L.

^a^ Pooled sample of six spot urine sample from six consecutive days in GW 18 and 36. One spot urine sample 3- and 6-months post-partum.

^b^ Log2 transformed values of TSH due to skewed data. Covariates in adjusted model: None

^c^ Covariates in adjusted model: Pre-pregnancy BMI and ferritin concentration

^d^ Covariates in adjusted model: Pre-pregnancy BMI and ferritin concentration

^e^ Dichotomous variable: 0= Reference category, normal thyroid function. 1= Disturbed thyroid function (TSH and/or fT4 or fT3 outside reference ranges). Coefficient given as odds ratio (OR). Covariates in adjusted model: Pre-pregnancy BMI.

CI, confidence interval; GEE, generalized estimating equations; GW, gestational week; TSH, thyroid stimulating hormone; fT3, free tri-iodothyronine; fT4, free thyroxine; UIC, urinary iodine concentration;

**Supplementary Table 3** TSH (mIU/L) by categories of timing of iodine-containing supplement (from pre-pregnancy until GW 18) in GEE models of repeated measurements during pregnancy (GW 18 and 36) and post-partum (3 and 6 months)

|  | **TSH (mIU/L)** ^b^ | | |
| --- | --- | --- | --- |
| **Use of supplements** ^a^ | β  (95% CI) | Estimated means (95% CI) | *P* |
| None (ref) | - | 1.68 (1.55-1.81) | - |
| Pre-pregnancy | -0.502  (-1.649, 0.644) | 1.18 (0.54-2.61) | 0.391 |
| GW 0-18 | -0.168  (-0.473, 0.136) | 1.49 (1.23-1.81) | 0.279 |
| Pre-pregnancy and GW 0-18 | -0.311  (-0.576, -0.047) | 1.35 (1.14-1.59) | 0.021 |

GEE models in exchangeable correlation matrix. TSH is entered with normal distribution with identity link function. Data are only presented as unadjusted models due to no covariates were selected after purposeful selection of covariates.

^a^ Supplement user defined as taking an iodine containing supplements >2 times/week

^b^ Log2 transformed values of TSH due to skewed data. Estimated means (95% CI) are anti-log2 for interpretation.

GW, gestational week

**Supplementary Table 4** fT3 (pmol/L) by categories of timing of iodine-containing supplement (from pre-pregnancy until GW 18) in GEE models of repeated measurements during pregnancy (GW 18 and 36) and post-partum (3 and 6 months)

|  | **fT3 (pmol/L)** | | | | | |
| --- | --- | --- | --- | --- | --- | --- |
|  | Unadjusted | | | Adjusted ^b^ | | |
| **Use of supplements** ^a^ | β  (95% CI) | Estimated means (95% CI) | *P* | β  (95% CI) | Estimated means (95% CI) | *P* |
| None (ref) | - | 4.29 (4.20-4.38) | - | - | 4.28 (4.17-4.39) | - |
| Pre-pregnancy | 0.220  (-0.115, 0.555) | 4.51 (4.19-4.83) | 0.198 | 0.163  (-0.570, 0.249) | 4.45 (4.14-4.76) | 0.328 |
| GW 0-18 | 0.393  (0.095, 0.692) | 4.68 (4.40-4.97) | 0.010 | 0.135  (-0.112, 0.382) | 4.42 (4.22-4.62) | 0.284 |
| Pre-pregnancy and GW 0-18 | 0.251  (0.083, 0.419) | 4.54 (4.40-4.68) | 0.003 | 0.199  (0.014, 0.384) | 4.48 (4.34-4.63) | 0.035 |

GEE models in exchangeable correlation matrix. fT3 is entered with normal distribution with identity link function.

^a^ Supplement user defined as taking an iodine containing supplements >2 times/week

^b^ Covariates in adjusted model: Pre-pregnancy BMI and ferritin concentration.

**Supplementary Table 5** fT4 (pmol/L) by categories of timing of iodine-containing supplement (from pre-pregnancy until GW 18) in GEE models of repeated measurements during pregnancy (GW 18 and 36) and post-partum (3 and 6 months)

|  | **fT4 (pmol/L)** | | | | | |
| --- | --- | --- | --- | --- | --- | --- |
|  | Unadjusted | | | Adjusted ^b^ | | |
| **Use of supplements** ^a^ | β  (95% CI) | Estimated means (95% CI) | *P* | β  (95% CI) | Estimated means (95% CI) | *P* |
| None (ref) | - | 14.5 (14.1-14.9) | - | - | 14.4 (14.0-14.8) | - |
| Pre-pregnancy | 1.097  (-0.414, 2.608) | 15.6 (14.1-17.1) | 0.155 | 0.988  (-0.534, 2.510) | 15.4 (13.9-16.9) | 0.203 |
| GW 0-18 | 0.264  (-0.697, 1.225) | 14.8 (13.9-15.7) | 0.590 | -0.084  (-1.258, 1.090) | 14.3 (13.3-15.3) | 0.888 |
| Pre-pregnancy and GW 0-18 | 0.889  (0.213, 1.565) | 15.4 (14.8-16.0) | 0.010 | 0.825  (0.107, 1.543) | 15.2 (14.7-15.8) | 0.024 |

GEE models in exchangeable correlation matrix. fT4 is entered with normal distribution with identity link function.

^a^ Supplement user defined as taking an iodine containing supplements >2 times/week

^b^ Covariates in adjusted model: Pre-pregnancy BMI and ferritin concentration.

**Supplementary Table 6** fT4/fT3 ratio by categories of timing of iodine-containing supplement (from pre-pregnancy until GW 18) in GEE models of repeated measurements during pregnancy (GW 18 and 36) and post-partum (3 and 6 months)

|  | **fT4/fT3 ratio** | | | | | |
| --- | --- | --- | --- | --- | --- | --- |
|  | Unadjusted | | | Adjusted ^b^ | | |
| **Use of supplements** ^a^ | β  (95% CI) | Estimated means (95% CI) | *P* | β  (95% CI) | Estimated means (95% CI) | *P* |
| None (ref) | - | 3.41 (3.24-3.52) | - | - | 3.41 (3.30-3.51) | - |
| Pre-pregnancy | 0.068  (-0.161, 0.296) | 3.48 (3.28-3.52) | 0.562 | 0.084  (-0.148, 0.316) | 3.49 (3.28-3.70) | 0.476 |
| GW 0-18 | -0.232  (-0.404, -0.060) | 3.18 (3.05-3.31) | 0.008 | -0.143  (-0.324, 0.038) | 3.26 (3.12-3.41) | 0.122 |
| Pre-pregnancy and GW 0-18 | -0.004  (-0.203, 0.196) | 3.41 (3.24-3.58) | 0.972 | 0.015  (-0.185, 0.214) | 3.42 (3.26-3.59) | 0.886 |

GEE models in exchangeable correlation matrix. fT4/fT3 ratio is entered with normal distribution with identity link function.

^a^ Supplement user defined as taking an iodine containing supplements >2 times/week

^b^ Covariates in adjusted model: Pre-pregnancy BMI and ferritin concentration
